# Forecasting maldistribution of human resources for healthcare and patients in Japan: a utilization-based approach

**DOI:** 10.1186/s12913-019-4470-x

**Published:** 2019-09-09

**Authors:** Tomoki Ishikawa, Yuji Nakao, Kensuke Fujiwara, Teppei Suzuki, Shintaro Tsuji, Katsuhiko Ogasawara

**Affiliations:** 1 Institute for Health Economics and Policy, No.11 Toyo-kaiji Bldg,1-5-11, Nishi-Shimbashi, Minato-ku, Tokyo, 105-0003 Japan; 2 Faculty of Health Sciences, Hokkaido University, N12W5, Kita-ku, Sapporo, 060-0812 Japan; 3 KKR Sapporo Medical Center, Hiragishi 1 jo 6 chome, Toyohira-ku, Sapporo, 062-0931 Japan; 4 Hokkaido University of Education, Iwamizawa, Midorigaoka, Iwamizawa, Hokkaido 068-8642 Japan

**Keywords:** Forecasting, Demand-supply balance, Physician shortage, Maldistribution, Utilization-based approach

## Abstract

**Background:**

Hokkaido’s demographic trend of population decrease with aging population is remarkable even in Japan. Although healthcare policy decision-makers need to appropriately allocate resources while grasping regional demands, not much is available on whether medical demand would increase or not for future. In addition, little is known about what impact will current situation have on future demand-supply balance and equality by regions. This study aims to support decision-making in human resource planning for coping with changing population structure by forecasting future demand, and evaluation those regional maldistributions.

**Method:**

We set patients with acute myocardial infarction or cerebral stroke, and all medical care as study subjects and analyzed for 2015, 2025, and 2035 in Hokkaido and each Secondary Medical Care Area. We used a utilization-based approach to estimate the healthcare supply–demand balance in the future. Moreover, we evaluated the regional maldistribution of demand-supply balance by calculating Herfindahl-Hirschman Index, Gini Coefficients, the number of physicians/specialists per patient. Moreover, we conducted sensitivity analysis to evaluation impact on aspects of demand-supply balance by uncertainty of utilization for future.

**Results:**

Our results displayed that concentration of patients will progress, while regional distribution will shrink in all subject. However, from comparison based on all medical care, Gini Coefficients of acute myocardial infarction and cerebral stroke has always been high. This suggest that the resource allocation of them has room for improvement. In addition, our analysis showed the change in this balance will differ in each region in the future. Moreover, demographic change will not consistent with the number of patient change from 2015 to 2035.

**Conclusion:**

These results suggest policy planners should use the number of patient by disease, by region as indicator of demand, instead of provider-to-population ratios being in use today. The result of our sensitivity analysis show two findings. First, the range of each indicator have possible for future. Second, increase of utilization, for instance lowing barrier in the use by development operation of patient transportation in AMI/CS, would improve maldistribution of opportunity for resident to get emergency medical services.

## Background

### Japanese demographic change, and characteristic of Hokkaido

**Fig. 1:**
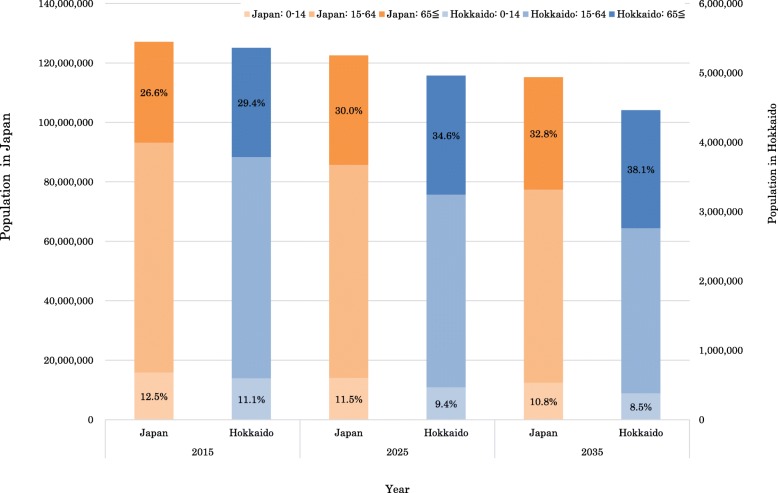
Population projection by age in Japan, Hokkaido [2]

Healthcare systems in developed countries are facing the challenge of dealing with changing social structures resulting from rapidly aging populations [1]. Japan has particularly notable problems with its declining birth rate and corresponding population aging. Figure 1, created with reference to the National Institute of Population and Social Security Research (IPSS), shows this trend will continue [2]. Japan’s total population is projected to decrease from 127,094,745 in 2015 to 115,215,698 by 2035 (rate: − 9.35%). In terms of age groups, the population < 15 years old will decline by 21.87% from the 2015 figure, while those aged ≥65 will increase by 11.66%. The population aging rate (those aged ≥65 among the total population) will reach 32.8% by 2035, and concern exists over how this will influence disease distribution. The IPSS also projected trends in demographic change will vary from region to region.

**Fig. 2:**
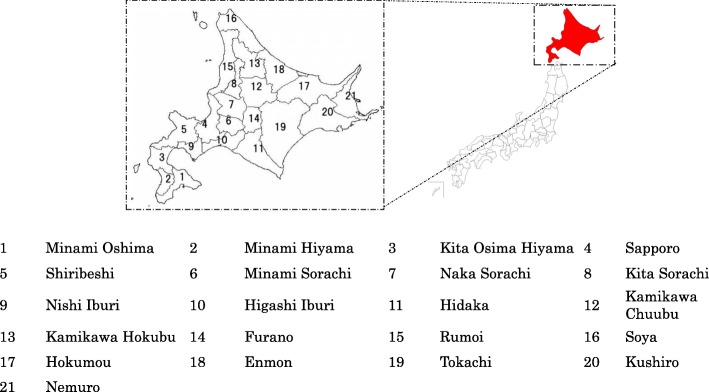
Location of Hokkaido and 21 SMCAs

Moreover, Hokkaido, which is the northernmost prefecture in Japan and has the largest area and lowest population density of all prefectures (Fig. 2), serves as an example. Figure 1 also shows, the trend demographic change in Hokkaido. Hokkaido’s population is predicted to decrease and be aging by 2035. Additionally, in Hokkaido in 2010, 34.8% of the population was distributed in the prefectural capital city of Sapporo, when a predicted 40.8% of the population will be concentrated in Sapporo in 2040 [3]. The progressive aging will necessitate improvement of the healthcare provision system because aging will lead to increased morbidity and mortality rates related to both chronic and acute diseases [4]. In fact, elder people utilize health services than young people, some previous study analyzed health care utilization patterns focused on specific diseases or services [5, 6].

These suggest that aging caused increasing of utilization healthcare services, it would require to increase medical supply quantity. In contrast, decreasing population would result in shrink of medical services market in itself. Not much is available on whether medical demand will increase or decrease, and how much influence it will have on what disease domain, considering aging population and population decrease at the same time.

In addition, population concentration requires rebuilding not only for healthcare institutions and equipment, but also for allocation of human resources in accordance with particular local healthcare needs. Therefore, healthcare policy decision-makers need to appropriately allocate healthcare resources while grasping individual change of regional demands and influence for demand by both of aging and concentration. In other words, future changes in supply-demand balance for each region have not been clarified. To summarize above, in Japan, it is still incompletely understood whether medical demand would increase or decrease by region by disease domain. What is more, little is known about proper measures for medical resources allocation to deal with future change of demand quantity. In Japan, the shortage of physicians has been recognized as a major medical issue. According to “Survey of Doctors, Dentists, and Pharmacists” which operating by the Ministry of Health, Labour and Welfare (MHLW) every 2 years, the number of physicians in Japan has been increasing and the growth rate from 2000 to 2014 was 18.1% [3]. As well as this, the number of physicians per 1000 residents is growing and was 2.34 in 2014. However, MHLW has reported that inadequate numbers of physicians result from absolute shortage and maldistribution by region [7].

Hokkaido is as well as an area noted for physician shortages and regional maldistribution. The number of physicians per 100,000 people in 2014 was 233.6 in Japan and 230.6 in Hokkaido, illustrating Hokkaido’s shortfall. Apart from the shortage, about 90% of physicians in Hokkaido are concentrated in urban areas, with about half in Sapporo [8]. In the same way, in terms of the number of physicians per 100,000 people by Secondary Medical Care Area (SMCA; administrative area encompassing several municipalities) in Hokkaido, only the two regions of Sapporo (281.2) and Kamikawa Chubu (320.5) surpass the national average (233.6). From view of allocation for human resources, Hokkaido faces a more serious situation than Japan as a whole. Hokkaido has issues of shortage and concentration of physician, since this is also related to problem on demand side, the supply-demand balance evaluation which analyzed both simultaneously is needed to propose appropriate and evidence-based political measures.

Japan’s Medical Care Act mandates that each prefecture formulate a Medical Care Plan every 5 years so as to establish a medical provision system with a SMCA as a regional unit [9]. The plan is intended for appropriately allocating healthcare resources, with the SMCAs functioning as geographical units that provide and manage most healthcare-related services. The plan also promotes activities geared toward addressing five diseases—cancer, cerebral stroke (CS), acute myocardial infarction (AMI), diabetes and psychiatric disorders—and five project focuses—emergency medical care, disaster medical care, remote healthcare, perinatal care, and pediatric medical care. For CS and AMI, from an emergency medical service standpoint, an appropriate medical provision system is vitally important. Older people also have higher incidence of stroke and cardiovascular diseases, and there is increasing need to develop a medical provision system to address these in Hokkaido.

MHLW and Ministry of Education, Culture, Sports, Science and Technology are planning measures such as increasing the number of training physicians, but shortages and maldistribution are expected to continue in Hokkaido. Human resource planning for healthcare is generally concerned with ensuring availability of an adequate number and type of health human resources to deliver the right alignment of services, recipients, and timing. Therefore, policy-making needs to be based on objective predictions of future medical supply–demand balance.

The following is a summary of the above. Because few Japan studies have reported on the population change’s impact on the supply–demand balance. Appropriate and evidence-based policy planning with regard to healthcare human resources must be based on decisions informed by objective prediction data. However, impact on medical demand by change demographic for future has not been clarified. This study aims to support decision-making in human resource planning for coping with changing population structure by picking up Hokkaido, facing severe situation, as a trial region. We used a utilization-based approach to analyze the healthcare supply–demand balance estimated into the future.

On forecasting method of medical demand-supply balance, in recent years, a medical need-based analytical framework has been proposed and applied as an approach for evaluating the balance between supply and demand [10]. Meanwhile, other studies have claimed that need-based planning of health human resources leads to excess supply and to inefficiency [11]. Although there are pros and cons for these methods, at present, utilization-based approach is often adopted in previous research. Besides, this study focus on Japanese demographic change, we should adopt a traditional, utilization-based approach employing [12].

## Methods

### Geographical units of analysis

We chose Hokkaido as our study target (Fig. 2). Hokkaido’s area is about 83,000 km^2^, or approximately 22% of the total area of Japan. On the other hand, as mentioned above, Hokkaido has the lowest population density in Japan, and Hokkaido’s population is largely concentrated in the city of Sapporo [3]. At the same time, depopulation continues in the rural areas that make up most of the prefecture. Therefore, there is great need for medical care plan that considers optimal production of services and allocation of resources [1]. Hokkaido has 21 SMCAs that encompass several municipalities. We analyzed both Hokkaido overall and each SMCA in the prefecture.

### Data collection

We obtained data tables based on surveys from the Japanese governmental e-Stat portal and from the IPSS [2, 3, 13]. The number of physicians (all clinicians and specialists) for each SMCA, future and present populations, and rates of estimated patients by disease classification, other variables selected for analysis were collected from assorted surveys, including the Patient Survey; Survey of Physicians, Dentists, and Pharmacists; national census; and relevant society databases in Japan (Table 1) [3, 4, 18]. In this study, we calculation some indicator or estimated by using these public data without our own original survey.

**Table 1:** Data source and those difinition for construction of forecasting model

Variables	Definition	Sources	Publication year	Responsible organization
Supply side				
The number of physicians/specialist in the future	Futre population each SMCA × physicians/Specialist number per population in 2015	Population projection for Japan [2], The Survey of Physicians, Dentists, and Pharmacists [3]	2014,2015	National Institute of Population and Social Security Research, Ministry of Health, Labour and Welfare
Future Population SMCA	Future Population data at 2025,2035	Population projection for Japan [2]	2015	National Institute of Population and Social Security Research
The number of physicians	Extraction only clinical physician	The Survey of Physicians, Dentists, and Pharmacists [3]	2014	Ministry of Health, Labour and Welfare
The number of physician in each SMCA	Extraction only clinical physician	Annual Health Report in Hokkaido [14]	2014	Hokkaido government
The number of specialist for treatment CS	The number of specialist engaged in neurosurgery	List of certified specialists [15]	2017	the Japan Neurosurgical Society
The number of specialist for treatment AMI	The number of specialist engaged in cardiovascular	List of certified specialists [16]	2017	the Japan Circulation Society
Location of each specialist to treat AMI/CS	Public medical institution responsible for acute care	Medical care plan in 2016 [17]	2016	Hokkaido government
Population in Hokkaido/each SMCA	Population in Hokkaido/each SMCA	National Census [4]	2015	Statistics Bureau
Demand side				
The number of patinet by disease/SMCA	Future Population by age/sex/SMCA × Utilization rate at 2015 by age/sex/SMCA/disease	Population projection for Japan [2], Patient Survey [10]	2014,2015	National Institute of Population and Social Security Research, Ministry of Health, Labour and Welfare
Future Population by age/sex/SMCA	Future Population data at 2025,2035	Population projection for Japan [2]	2015	National Institute of Population and Social Security Research
Utilization rate by classification of disease	Utilization rate by classification of disease	Patient survey [10]	2014	Ministry of Health, Labour and Welfare

### Literature review of evidence-based approach to medical demand

Forecasting of health workforce required to meet demand has been difficult task. There are some types of forecasting approaches, and they are so not well-defined that can cause confusion in planning.

The simplest approach is the way using the workforce-to-population ratio for determining the workforce required to serve a given population. This approach considered typically demographic data such as population growth. Cromwell et al. reported that school enrollments is needed to fill needs given the predicted growth in the United States anesthesia services by using the workforce to population ratio [19]. The workforce-to population ratio is also called provider-to-population ratios (PPR). Fields et al. demonstrated that illustration relationship between PPR, rurality and population health by counties unit of alalysis [20]. In Japan, health care planning by using PPR as indicator of resources allocation have been daily used [6]. However, some studied have pointed out that estimation by using only PPR required additional analysis, because this approach is likely to deal with the matter of unequal distribution of healthcare workforce [21]. J Bauer et al. claimed that PPRs don’t reveal detailed spatial variations within an area nor taking into account for boundary crossing of patients and physicians [22].

Therefore, although discussion progresses on methods for estimating demand in medical care, we adopted this approach in this research design, in consideration of the availability of the data according to the previous study.

### Study design

**Fig. 3:**
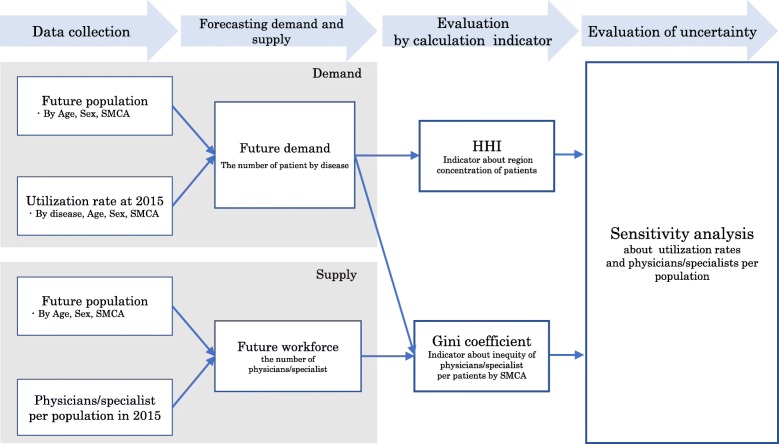
Conceptual scheme of this study design

We designed the present study from the perspective of securing equitable opportunity to receive medical services. Our overall study procedure consisted of five sub-procedures. The first was forecasting medical demand by a utilization-based approach. The second was forecasting physicians/specialists as analyzed from the standpoint of population change. The third step was evaluation of influence of supply–demand balance change on spatial distribution, by calculation of Gini coefficients (GC). The fourth, we estimated the concentration of patients in the market by calculating the Herfindahl-Hirschman Index (HHI), a widely accepted measure in antitrust enforcement. Finally, we conducted a sensitivity analysis to estimate the influence of the uncertainties of some variables. Figure 3 shows the model methodology used in this study. Table 2 shows what we assumed in predictive analysis using models.

**Table 2:** Key assumptions of forecasting demand and supply

Supply side	
Allocating physicians according to demographic change in each SMCA	
Productivity per a physician will unchange over time	
Health care system and medical insurance sytem will remain	
Demand side	
Patient behavior by disease will not change	
Demand increasing by innovation of technology, such as in screening, will not occur	
The accessibility level to medical services wil remain constant	
Working scope of medical stuff will remain as it is	
Age and sex specific levels health will remain constant over time	

#### Forecasting medical demand based on utilization-based approach

In a utilization-based approach, future demand is calculated by multiplying future population by utilization rate by disease. First, we obtained utilization rate by disease and population data as variables, by municipality and by age and sex in the future from “Patient survey” by the MHLW and “Population Projections for Japan” by the IPSS [2, 18]. We then multiplied these variables for future estimates of the number of patients as the criterion for demand in each municipality. Next, we aggregated these numbers of patients by SMCA. We conducted this procedure on AMI, CS, and all medical care (AMC). To provide further details, we took both inpatients and outpatients as study subjects and analyzed for 2015, 2025, and 2035.

#### Forecasting physicians/specialists for analysis

To start, to project future physician/specialists numbers, we collected population figures and the numbers of patients/physicians certified as specialists, by each SMCA, for each specialty, for 2015. We then calculated the number of physicians/specialists per person in Hokkaido. To focus on demographic change, we assumed the population-to-physician/specialists ratio would be constant from 2015. Following this assumption, learning a traditional method called the supply projection approach, we estimated the number of physicians/specialists in 2025 and 2035 by multiplying the future population by physicians/specialists per people in 2015 for each SMCA and specialty [23–25].

#### Calculating GC of physician per patients

The GC is widely used to measure inequity in income distribution. Several research groups have applied this method to evaluate health resource distribution; for example, physicians or medical facilities [26–28]. We adopted the GC as an indicator to refer to regional maldistribution of physicians/specialists to aid evaluation of inequity of human resources by SMCA. GC was determined as:

$$ \mathrm{G}=\left(\sum \limits_{i=1}^{n-1}{X}_i\times {Y}_{i+1}\right)-\left(\sum \limits_{i=1}^{n-1}{X}_{i+1}\times {Y}_i\right) $$

where *G* is the *GC*, *n* is the number of SMCAs, *Xi* is the cumulative percentage of the ratio of patients in the area of SMCA *i*, and *Yi* is the cumulative percentage of the ratio of physicians. The GC ranges from 0 to 1, where, theoretically, 0 is perfect equity and 1 is perfect inequity. In field of human resources for healthcare, as we know, there are no absolute standards for evaluating GC. Thus, we discussed GC focusing on relative change over time.

#### Calculating HHI

The HHI has been used to evaluate mergers and acquisitions, but has also been applied to include concentration of health care resources [29–32]. It is calculated by summing the squares of each firm’s market share. In this study, the “share” is the proportion of the number of patients for each SMCA. The HHI ranges from 0 to 1, where 1 is a market monopoly, while approaching 0 indicates a market with a larger number of equal competitors. Noticeably, in fact, the HHI never reaches 0, with the lower limit being 1/N, where N is the number of firms in the market. For this study, HHI was interpreted as the measure of patient concentration, so as to forecast future demand transfer. As is standard, we considered markets highly concentrated if their HHI was > 0.250, moderately concentrated if 0.150–0.250, not concentrated if 0.100–0.150, and highly competitive if < 0.100 [32].

#### Sensitivity analysis

Our forecasting by utilization-based approach rely on assumption that current situation will not change for future: health care policy, patient behavior, physician per population, and performances by physicians. However, as a matter of course, some factors have uncertainty in future. Uncertainty must be assessed so that policy planners can anticipate possible variations and adapt the planning of human resources. In particular, demand volume would be affected by change of many factor; new model of care, policy change, development infrastructure for access healthcare, and so on. We conducted sensitivity analysis to evaluate the impact of the uncertainties of utilization rate on GC and HHI. We assumed that future utilization variation occurs within the range of past trend. The range of utilization rate was calculated by average of utilization rate from past 15 years, conducted 5 times national Patient Survey by MHLW, we set these number as the range of sensitivity analysis correspond following scenarios: decreasing (Scenario A) or increasing (Scenario B) the utilization rate per 100,000 population from the current number by AMI: 2 point, CS: 71 point, AMC: 630point.

## Results

**Fig. 4:**
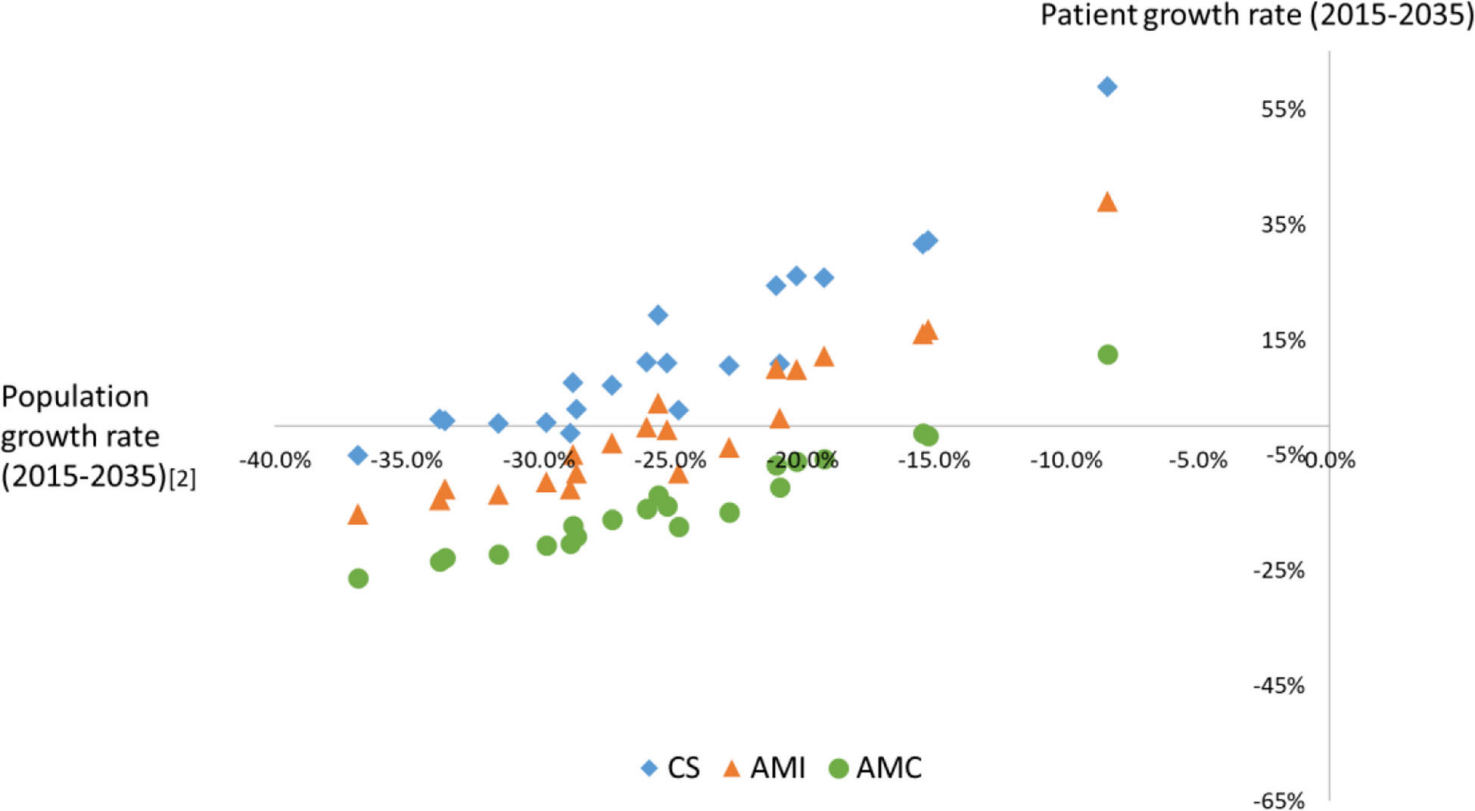
The relationship between population and patient growth rate from 2015 to 2035 in each SMCA

Table 3 shows forecasting results of the number of physicians/specialist and Table 4 displays forecasts of the number of patients in each SMCA. Figure 4 shows the relationship between population growth rate and patient growth rate from 2015 to 2035 in each SMCA. We also summarized using the number of physicians per patient (Table 5). The number of physicians in Hokkaido was forecast to decrease respectively (rates, cardiovascular specialists: − 21.8%, neurosurgical specialists: − 21.8%, all clinical physicians: − 24.4%). Similarly, the number of AMC patients would decrease by 2025. However, the number of patients treated for CS and AMI respectively was projected to increase by 2025. These outcomes result in lower numbers of physicians per patients for each condition (rate, CS: − 40.8%, AMI: − 32.9%, AMC: − 23.1%). The Medical Care Act stipulates hiring one physician for 40 patients per hospital as a placement standard for outpatient medical care; i.e., this criterion is 0.025 in terms of physicians per patients, and data that did not reach one were shown in gray for AMC in Table 5. This standard cannot be applied for each specialist. This suggests that the number of physicians per patient may fall below the placement standard between 2015 and 2025.

**Table 3:** Forecasted physicians, 2015 to 2035

SMCA in Hokkaido	Cardiovascular specialist			Neurosurgical specialist			All clinical physician		
	2015	2025	2035	2015	2025	2035	2015	2025	2035
Minami Oshima	17	14	13	16	13	13	904	728	684
Minami Hiyama	0	0	0	0	0	0	32	26	24
Kitaoshima Hiyama	0	0	0	1	1	1	48	39	36
Sapporo	140	117	109	134	112	105	6813	5486	5151
Shiribeshi	5	4	4	7	6	5	428	345	324
Minami sorachi	6	5	5	3	2	2	293	236	222
Naka sorachi	5	4	4	7	6	5	255	205	193
Kita sorachi	0	0	0	1	1	1	72	58	54
Nishi iburi	6	5	5	6	5	5	420	338	318
Higashi iburi	2	2	2	9	7	7	350	282	265
Hidaka	0	0	0	0	0	0	75	60	57
Kamikawa chubu	25	21	20	24	20	19	1310	1055	990
Kamikawa hokubu	3	2	2	3	2	2	115	93	87
Furano	0	0	0	0	0	0	57	46	43
Rumoi	0	0	0	1	1	1	74	60	56
Souya	0	0	0	2	2	2	66	53	50
Hokumou	6	5	5	8	7	6	358	288	271
Enmon	4	3	3	0	0	0	603	486	456
Tokachi	13	11	10	15	12	12	402	324	304
Kushiro	7	6	5	14	12	11	100	81	76
Nemuro	2	2	2	0	0	0	78	63	59
Hokkaido	241	201	188	251	209	196	12,853	10,349	9718

**Table 4:** Forecasted patients by disease, 2015 to 2035

SMCA in Hokkaido	Cerebral stroke			Acute myocardial infarction			All medical care		
	2015	2025	2035	2015	2025	2035	2015	2025	2035
Minami Oshima	1140	1260	1265	299	310	297	29,580	28,341	25,505
Minami Hiyama	87	90	83	22	22	19	2029	1804	1494
Kitaoshima Hiyama	135	140	134	34	33	31	3145	2874	2508
Sapporo	5752	7710	9139	1540	1917	2139	165,399	181,746	186,181
Shiribeshi	726	763	730	187	185	169	17,589	16,183	13,973
Minami sorachi	586	642	631	151	153	144	13,954	13,102	11,559
Naka sorachi	400	424	402	102	101	90	9290	8487	7227
Kita sorachi	132	142	134	33	33	29	2910	2647	2232
Nishi iburi	614	687	678	160	166	154	15,245	14,607	12,974
Higashi iburi	548	665	724	148	168	173	15,343	15,768	15,098
Hidaka	215	232	230	56	57	54	5437	5114	4561
Kamikawa chubu	1183	1407	1491	312	344	343	30,518	30,699	28,630
Kamikawa hokubu	230	245	237	59	59	54	5485	5117	4532
Furano	131	144	145	34	35	35	3335	3216	2983
Rumoi	174	184	176	45	44	40	4082	3712	3150
Souya	204	225	227	54	56	54	5284	5040	4530
Hokumou	675	793	839	177	194	194	17,224	17,164	16,057
Enmon	242	258	249	62	62	57	5802	5376	4698
Tokachi	965	1165	1270	254	285	295	25,520	26,083	25,228
Kushiro	661	763	788	177	190	184	17,726	17,251	15,611
Nemuro	197	231	247	53	58	59	5568	5558	5254
Hokkaido	14,998	18,170	19,820	3958	4472	4614	400,465	409,889	393,986

**Table 5:** Forecasted physician per patients, 2015-2035

SMCA in Hokkaido	Cerebral stroke			Acute myocardial infarction			All medical care		
	2015	2025	2035	2015	2025	2035	2015	2025	2035
Minami Oshima	0.0149	0.0112	0.0105	0.0536	0.0429	0.0422	0.0306	0.0257	0.0268
Minami Hiyama	0.0000	0.0000	0.0000	0.0000	0.0000	0.0000	0.0158	0.0143	0.0162
Kitaoshima Hiyama	0.0000	0.0000	0.0000	0.0292	0.0251	0.0256	0.0153	0.0134	0.0145
Sapporo	0.0243	0.0151	0.0120	0.0870	0.0582	0.0490	0.0412	0.0302	0.0277
Shiribeshi	0.0069	0.0055	0.0054	0.0374	0.0315	0.0324	0.0243	0.0213	0.0232
Minami sorachi	0.0102	0.0078	0.0074	0.0199	0.0163	0.0163	0.0210	0.0180	0.0192
Naka sorachi	0.0125	0.0098	0.0097	0.0684	0.0579	0.0607	0.0275	0.0242	0.0267
Kita sorachi	0.0000	0.0000	0.0000	0.0299	0.0254	0.0268	0.0247	0.0219	0.0244
Nishi iburi	0.0098	0.0073	0.0069	0.0375	0.0301	0.0304	0.0275	0.0232	0.0245
Higashi iburi	0.0036	0.0025	0.0022	0.0608	0.0445	0.0407	0.0228	0.0179	0.0175
Hidaka	0.0000	0.0000	0.0000	0.0000	0.0000	0.0000	0.0138	0.0118	0.0124
Kamikawa chubu	0.0211	0.0148	0.0131	0.0770	0.0580	0.0548	0.0429	0.0344	0.0346
Kamikawa hokubu	0.0130	0.0102	0.0099	0.0509	0.0427	0.0433	0.0210	0.0181	0.0192
Furano	0.0000	0.0000	0.0000	0.0000	0.0000	0.0000	0.0171	0.0143	0.0144
Rumoi	0.0000	0.0000	0.0000	0.0224	0.0189	0.0196	0.0181	0.0160	0.0178
Souya	0.0000	0.0000	0.0000	0.0372	0.0298	0.0291	0.0125	0.0105	0.0110
Hokumou	0.0089	0.0063	0.0056	0.0453	0.0344	0.0322	0.0208	0.0168	0.0169
Enmon	0.0166	0.0129	0.0126	0.0000	0.0000	0.0000	0.1039	0.0903	0.0970
Tokachi	0.0135	0.0093	0.0080	0.0590	0.0438	0.0397	0.0158	0.0124	0.0120
Kushiro	0.0106	0.0076	0.0069	0.0792	0.0615	0.0595	0.0056	0.0047	0.0048
Nemuro	0.0102	0.0072	0.0063	0.0000	0.0000	0.0000	0.0140	0.0113	0.0112
Hokkaido	0.0161	0.0110	0.0095	0.0634	0.0467	0.0425	0.0321	0.0252	0.0247

It is forecasted that Hokkaido’s population would decrease from 2015 to 2035 in each SMCA, in Fig. 4, each point took 2nd/3rd quadrants. The ratio of the number of points taking in 2nd to in 3rd varied by disease.

**Fig. 5:**
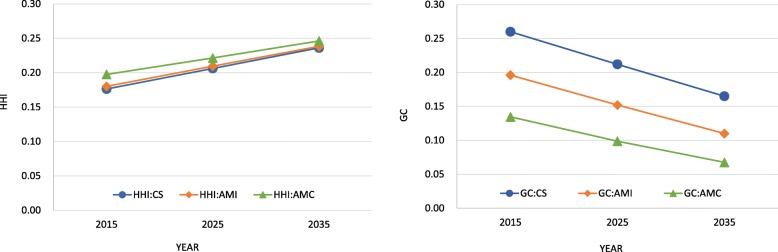
Forecasted HHI and GC

The resulting calculations of HHI and GC are shown in Fig. 5. These results showed that HHI will increase, yet GC will decrease. The HHI of AMC changed at relatively low rate, but showed a high trend in GC; i.e., AMI and CS would change with a relatively low HHI, while the GCs were forecast to change with a high HHI.

**Fig. 6:**
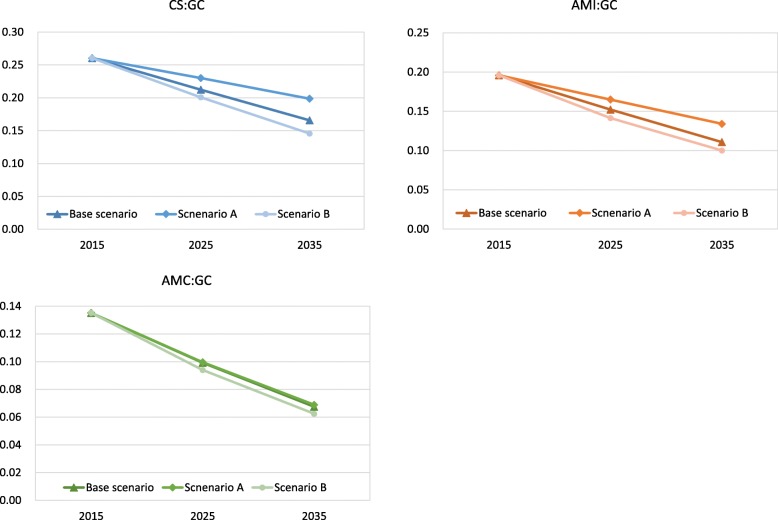
The results of sensitivity analysis on GC in CS, AMI, and AMC

**Fig. 7:**
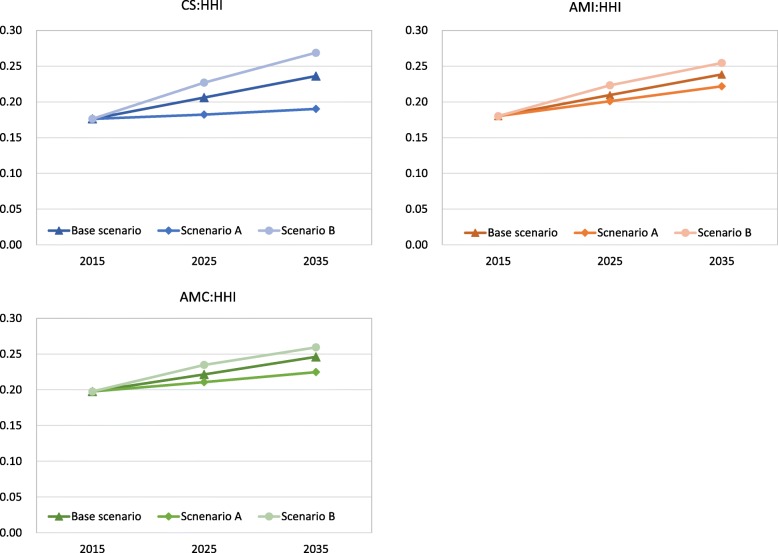
The results of sensitivity analysis on HHI in CS, AMI, and AMC

Our sensitivity analysis results are shown in Figs. 6 and 7. In scenario A (demand decreasing case), GC would be higher than base scenario, while HHI would be lower than it. Conversely, Scenario B (demand increasing case), GC would be lower than base scenario, while HHI would be higher than it. This trend was similarly observed in each disease.

## Discussion

Our forecasting results show difference of future trend by disease, it owe to reflection of a variation of utilization for each disease. This point is an advantage of forecasting demand based on a utilization-based approach.

Figure 4 visualized relationship population and patient growth rate by SMSAs. This result key suggestion that medical resource allocation plan considering only demographic change may have a risk of over/under estimation for future demand. This risk consist with disadvantage of the approach using only PPRs, as some previous studies claimed till now. From this reason, we propose that planners use indicator based on the number patient by disease/area in place of one based on PPRs as standard for allocation medical resource as suggestion in previous study [22]. Moreover, in the future, the degree of concentration of AMI and CS may parallel AMC or overtake it from result of calculation HHI. In addition, this suggests the possibility that regional maldistribution will shrink, but there is still room for improvement in AMI and CS, from view of comparison with AMC. Of particular note, the presence/absence of regional maldistribution cannot be evaluated from the absolute value of the GC, as it is a relative value. For this reason, we used GC only as an index representing the change of state through its increase/decrease.

Nevertheless, a shortage of physicians is already regarded as a problem in Japan, Table 5 shows the problem would be worsen. Furthermore, the Medical Care Act set out a placement standard for the number of physicians per outpatient to be 0.025. In our result for AMC, the model forecast this will fall below that standard between 2025 and 2035; this furthers concern about this impending depletion.

Many countries are interested in the relationship between demographic change and medical needs. Several studies have analyzed, in particular, the relationship with the placement of professions, especially since the development of human resources takes long time [14, 15, 33]. In any country, demographic trend differ from transition of healthcare workforce, these trend vary by area and by disease. This suspect also was observed in present study, it is effective that each region independently predicts the future trend. In Japan, each prefecture have to make a Medical Care Plan about approach by disease, such as “Cerebral Stroke” “Acute myocardial infarction”, it is better for planner to make a decision based on forecasting each area and each disease. This study approach is proposed as one of modeling method to estimate healthcare need and supply.

In recent years, several approach to estimate future health care needs is suggested, it is necessary to option appropriate method for purpose of researcher or health care policy planner. For example, the emphasis on planning of healthcare human resources has shifted away from a utilization-based approach toward a need-based one. Need-based approaches are based not only on medical utilization but also a host of factors such as new treatments and diagnostics, epidemiological changes, personal preferences, and socio-economic issues. Birch et al. forecast future requirements for the provider group using size, distribution, and levels of healthcare-related needs of the population [10].

Tomblin Murphy et al. applied a need-based approach toward resolving a nurse shortage in Canada [16]. Greenspoon et al. demonstrated the feasibility of applying an explicit, need-based model to resource planning in the area of radiation oncology [17]**.** However, other studies have proposed that need-based models divorce needs from socio-economic factors and personal preference, and that planning by this approach will bring about excess supply and inefficiency [11]. In fact, Tomblin Murphy reported utilization-based approaches, in which current or target rates of health service utilization are multiplied by estimates of future population, serve as the most widely used method in high-income Organisation for Economic Co-operation and Development (OECD) member countries [11]. Crettenden recently performed future projections for required medical workforce using utilization as a measure of demand [34]. McRae estimated a supply-and-demand model for general practitioner services in Australia [23].

Currently, utilization-based approaches have been often adopted in OECD countries in previous studies [11]. Further, the validity of applying a need-based approach in Japan is unclear from viewpoint of data availability, because variables required to model building don’t be collected in Japan at the national level.

In this study, we created forecasting model by collecting public survey data without our intervention. Therefore, variables imputed in the model is fairness and robustness is maintained. In our analysis procedure has high precision, because the input variables did not handle as statistical explanatory variables. However, in other words, this procedure cannot take into account probability of input variables for future. That is to say, there is limitation derived from setting assumptions in patient’s behaviors. We did not consider factors that affect medical supply and demand, such as changes in medical system, patient’s behavior, or doctor’s performance since utilization based approach focuses only on changes in the population. As a measures against this limitation, we conducted sensitivity analysis to estimate impact range on patient concentration and regional maldistribution of demand-supply balance by uncertainty of utilization. This results, looking from different view point, can be interpreted as a scenario analysis. As scenario A, if demand are decreasing, indicates would cause increase of GC and decrease HHI. In other words, this means that a decline of concentration and expansion of inequality. On the contrary, as scenario B, demand increasing would result in improvement inequality and progress of concentration. Tomblin have reported that the healthcare demand volume required is determined by population, health status, and level of services [12]. Among these factors, controllable one from healthcare policy is a level of services factor, for instance it includes accessibility in emergency medical; rapidly patient transportation to treatment by doctor car, development flight operations of helicopter emergency medical services in Japan. Our sensitivity analysis suggests that improvement accessibility by developing operating system patient transportation is one of solutions to secure opportunity equality for utilization of emergency medical service. We should have further argument about concrete operational plan by estimation for the volume of required human resources, and Cost-Effect analysis, to clearly indicate feasibility and usefulness in our next studies.

Healthcare policy decision-makers should maintain overall recognition of the future state of the medical supply–demand balance in order to develop suitable planning. Our analysis showed the change in this balance will differ in each region in the future, with the possibility of the physician shortage being aggravated, while maldistribution will shrink. Planners should use our objective analysis as a basis to implement countermeasures for regional maldistribution and shortages.

The present study, as with other studies using modeling methods, had analysis based on several assumptions, and had some limitations. Utilization-based approaches assume patients’ behavior will be unchanged from the present state. We conducted this analysis with a fixed value for the utilization rate. Because of this, our results will become more reliable after occurrence of certain events that affect patient behavior: innovation in early detection technology, progress of screening, and prevalence of preventive medicine. Such movements would enable us to update our model substituting newer rates to show more accurate results.

Moreover, we predicted changes in numbers of physicians based on population trends indicated in previous research. We consider this method valid for the purpose of this study to specify the impact of population change. The physician shortage will continue to worsen when allocating physicians in accordance with population change. However, a more accurate method of predicting the number of physicians is needed for perfectly predicting changes in supply and demand in the future. We have been studying a model for forecasting physician numbers using system dynamics [35]. We are considering from here forward combining our previous study with the methods in the present study to focus on more accurate supply and demand forecasts as a research subject.

This study focusses on supply–demand balance in the context of human resources. In fact, medical supply–demand balances should be evaluated not only in terms of human resources but also for accessibility, numbers of medical facilities and beds, and performance of each facility. Analysis of supply and demand in view of these various factors is a pertinent issue for carrying out a more detailed analysis that will be useful for supporting policy formulation. Increasing data collection on relevant values will minimize limitations in this area.

Also, building a model that takes into account not only changes in the population but also related factors will improve forecasts. Moreover, frequent model updating of data with regard to patients’ behavior will make forecasting results more reliable.

## Conclusions

In this study, we forecast future supply and demand of human resources in order to evaluate the level of patient concentration and regional maldistribution in SMCA units in Hokkaido, Japan. We demonstrated that a utilization-based approach can be used to forecast supply–demand balance in each SMCA and for individual diseases. This approach enabled several suggestions. First, patient concentration toward urban areas will proceed during the forecast period as evidenced by increasing HHI values. However, regional maldistribution will tend to improve, given the decreasing tendency of GC. We demonstrated the possibility of conducting analysis for supporting medical policies not only by evaluating patient concentration but also uneven distribution of physicians. Second, HHI and GC analysis showed that substantial maldistribution among CS and AMI, despite concentration of patients being lower than for AMC. By using AMC as a standard, we were able to relatively evaluate the status of each disease. Without absolute standards on maldistribution and concentration, we could relatively evaluate these statuses through comparison with AMC. Third, we should point out that resource allocation plan considering only the change of future population may have a risk of misleading for future demand. It is reasonable to support that our proposal that planners is better to use indicator based on population than to use one based on the number patient by disease/area as standard for allocation medical resource. Finally, our sensitivity analysis shows the variable range of GC and HHI in the future. It was also suggested that a development in accessibility would results in improvement of regional inequality.

This study suggests the necessity to construct a quantitative forecasting model. Thus, we believe that creating a model that takes into account future changes for some factors is a valuable endeavor.

## Data Availability

All the data used in this study were taken from on official statistics of Japan, and these data are publicly open data. Japanese National census and Fundamental statistics by MHLW are available for secondary analysis. Anyone can obtain these dataset from portal site of official Statistics of Japan: https://www.e-stat.go.jp/en. Population Projections for Japan data is available from following National Institute of Population and Social Security Research: https://www.e-stat.go.jp/en.

## Abbreviations

AMC: All medical care
AMI: Acute myocardial infarction
CS: Cerebral stroke
GC: Gini coefficient
HHI: Herfindahl-Hirschman Index
IPSS: National Institute of Population and Social Security Research
MHLW: Ministry of Health, Labour and Welfare
OECD: Organisation for Economic Co-operation and Development
SMCA: Secondary medical care area
